# Case Report: Long-Term Outcome Following Maximal Partial Resection in a Primary Pericardial Lymphangiohemangioma

**DOI:** 10.3389/fcvm.2021.706098

**Published:** 2021-07-22

**Authors:** Long Song, Chukwuemeka Daniel Iroegbu, Chengming Fan

**Affiliations:** Department of Cardiovascular Surgery, The Second Xiangya Hospital, Central South University, Changsha, China

**Keywords:** lymphangiohemangioma, case report, surgery, management, rare tumor

## Abstract

**Introduction:** Cardiac tumors are significantly rare, with pericardial lymphangiohemangioma amongst the rarest cardiac tumor types, with very few reported cases in the literature. Clinically, lymphangiohemangiomas are generally deemed unresectable due to their proximity to the myocardium and the uncertainty of the outcomes following subtotal resection.

**Case Report:** Herein, we report a case of a 40-year-old man diagnosed with a pericardial mass, dull thoracodorsal pain, and over a 10-years history of palpitation. Notably, the pericardial mass in the present case was found extended within the myocardium. Thus, a maximal safe resection was deemed preferable to a total resection. The surgically resected specimen showed pathological characteristics of a lymphangiohemangioma. After surgical resection of the lymphangiohemangioma, the patient was free of any tumor-related symptoms. Also, there was no evident tumor progression after a 4-year post-operative follow-up.

**Conclusion:** To the best of our knowledge, the present case study is the first in the literature to report on a long-term post-operative outcome following subtotal resection of a pericardial lymphangiohemangioma.

## Introduction

Tumors of the heart are significantly rare, with pericardial lymphangiomas amongst the rarest cardiac tumor types, with very few reported cases in the literature (1–3). Though both hemangiomas and lymphangiomas are classified under vascular tumors and vascular malformations, lymphangiomas are exceedingly rare, with the former making up ~5% of primary benign cardiac tumors (4). Cardiac tumors are characterized by endothelial-lined thin-walled spaces that contain lymph and disjointed native myocytes (2).

These benign neoplasms constitute a distinctive form of cardiac tumor primarily discovered in patients below 10-years old (2, 5, 6); however, they may appear or be detected later in life (1). Diagnosis based on imaging techniques is challenging, though the most helpful method seems to be magnetic resonance imaging (MRI) by elucidating the slow-flow components present in vascular malformations (7). Thus, the diagnostic approach is primarily surgical (1), given the increased risk of bleeding related to percutaneous biopsy (8).

Cardiac lymphangiohemangiomas may present in both intrapericardial and pericardial spaces, though benign, cardiac lymphangiohemangioma may cause arrhythmia, tamponade, heart failure, and myocardial ischemia. Therefore, complete excision of cardiac lymphangiomas is recommended if possible (6). It should be noted that Daubeney et al. reported intrapericardial lymphangioma, which spontaneously regressed 16-months after a pericardial window (9). Pericardial lymphangiohemangioma was first reported in 2014 with a surgical biopsy and was deemed to be unresectable (8).

Here we describe a 40-year-old man with pericardial lymphangiohemangioma who underwent a subtotal resection and was free of tumor-related symptoms and tumor progression after 4-years of follow-up.

## Case Report

A 40-years-old man was admitted to our institution with a 10-years history of chest pain and palpitation without significant weight loss. No headaches, fever, or cough was dictated with a body temperature of 36.3°C and blood pressure of 132/92 mmHg at resting conditions. Physical examination revealed that the pulse was 78 bpm. No additional heart sound or significant heart murmur was heard over the chest wall. There were no other notable clinical findings and family history of cardiovascular disease following medical history and physical examination. Also, laboratory tests were unremarkable.

**Figure 1:**
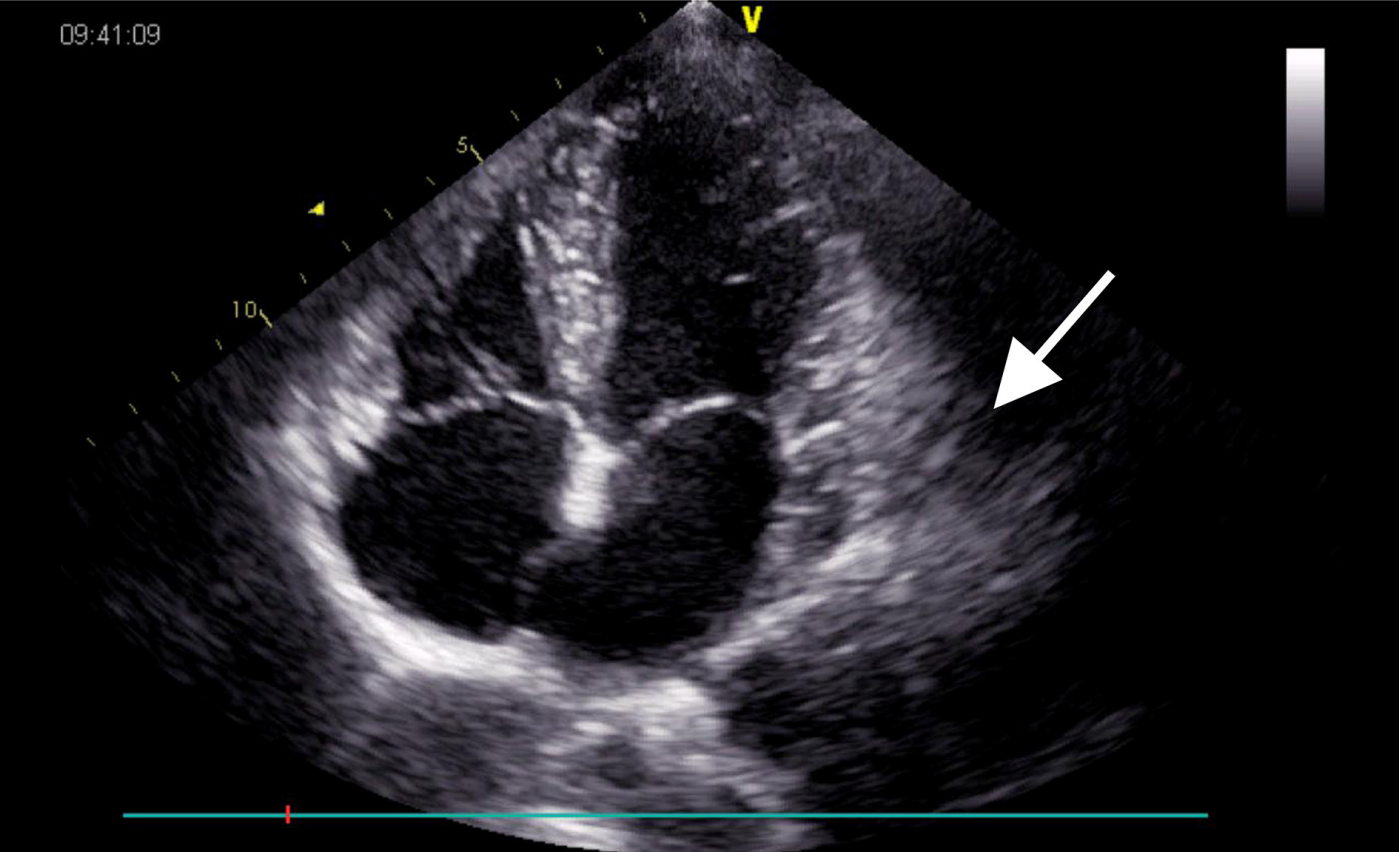
Echocardiogram pre-operatively showing a 32 × 66 mm heterogeneous mass (arrow) located in the left atrioventricular groove.

**Figure 2:**
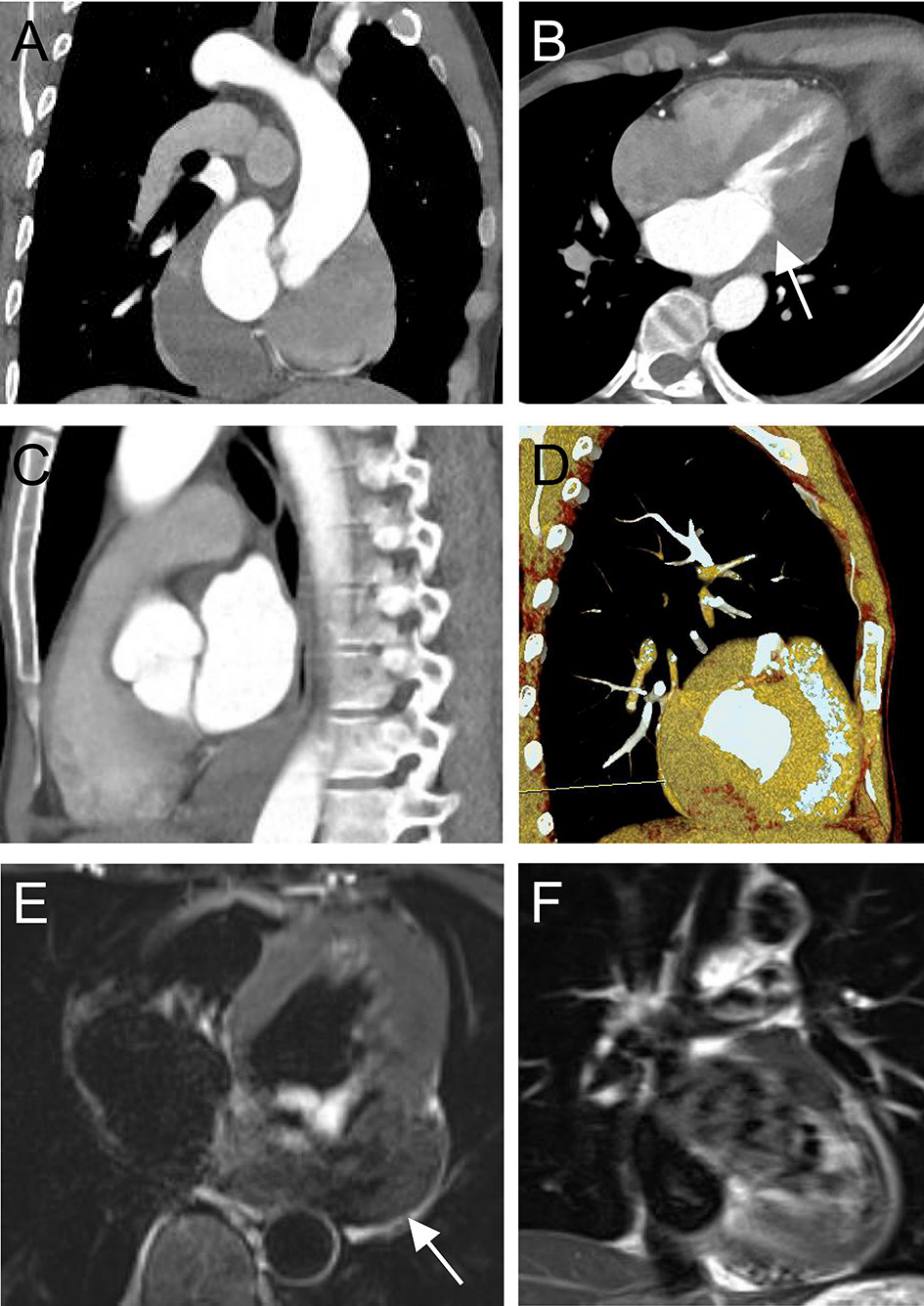
Computed tomography pre-operatively in coronal view **(A)**, four-chamber view **(B)**, sagittal view **(C)**, three-dimensional volume-rendered image, and **(D)** shows a tumor-like structure located in the left atrioventricular groove (arrow) with a fountain sign from the posterior wall of the left atrium to the abnormal mass. Cardiac magnetic resonance imaging **(E,F)** pre-operatively showing a heterogeneous mass with the posterior wall of the left ventricle involved.

Electrocardiography revealed sinus bradycardia without nodal block. No significant abnormalities were found following chest X-ray examination. Transthoracic echocardiography demonstrated normal ventricular function with a left ventricular ejection fraction of 69%; however, a saccular mass of 32 × 66 mm in size was discovered within the pericardial cavity, which extended toward the left atrioventricular groove. The saccular mass margins were well-defined and close to the left atrium and ventricle. The abnormal structure was heterogeneous with fractional acoustic enhancement and had gelatinous materials (Figure 1, arrow). Cardiac computed tomography (CT) showed a 54 × 50 mm mass that appeared like densely soft tissue with an oval shape margin. Simultaneously, a contrast-enhanced cardiac CT scan detected a fountain sign from the left atrium's posterior wall to the abnormal mass (Figures 2A–D, arrow). Cardiac MRI (Figures 2E,F, arrow) was also performed, which further confirmed the left ventricular posterior wall involvement as detected by the contrast-enhanced CT scan; nonetheless, no other metastatic mass was detected. Interestingly, the pre-operative coronary angiogram was normal.

The operative team evaluated the patient before the surgical excision of the tumor; afterward, a standard median sternotomy incision is performed. Following exploratory pericardiotomy, a botryoidal saccular mass was discovered below the left atrial appendage and above the left pulmonary vein with the size of about 10 × 8 × 6 cm. Due to the saccular mass size and its botryoidal texture, the patient's aorta was cannulated. Separate cannulas were placed in the superior vena cava and inferior vena cava. After full heparinization, cardiopulmonary bypass (CPB) was applied. The saccular tumor was filled with dark red liquid and a fibrous membranous network structure. Furthermore, the abnormal structure was found closely connected to the left atrium and left ventricle.

**Figure 3:**
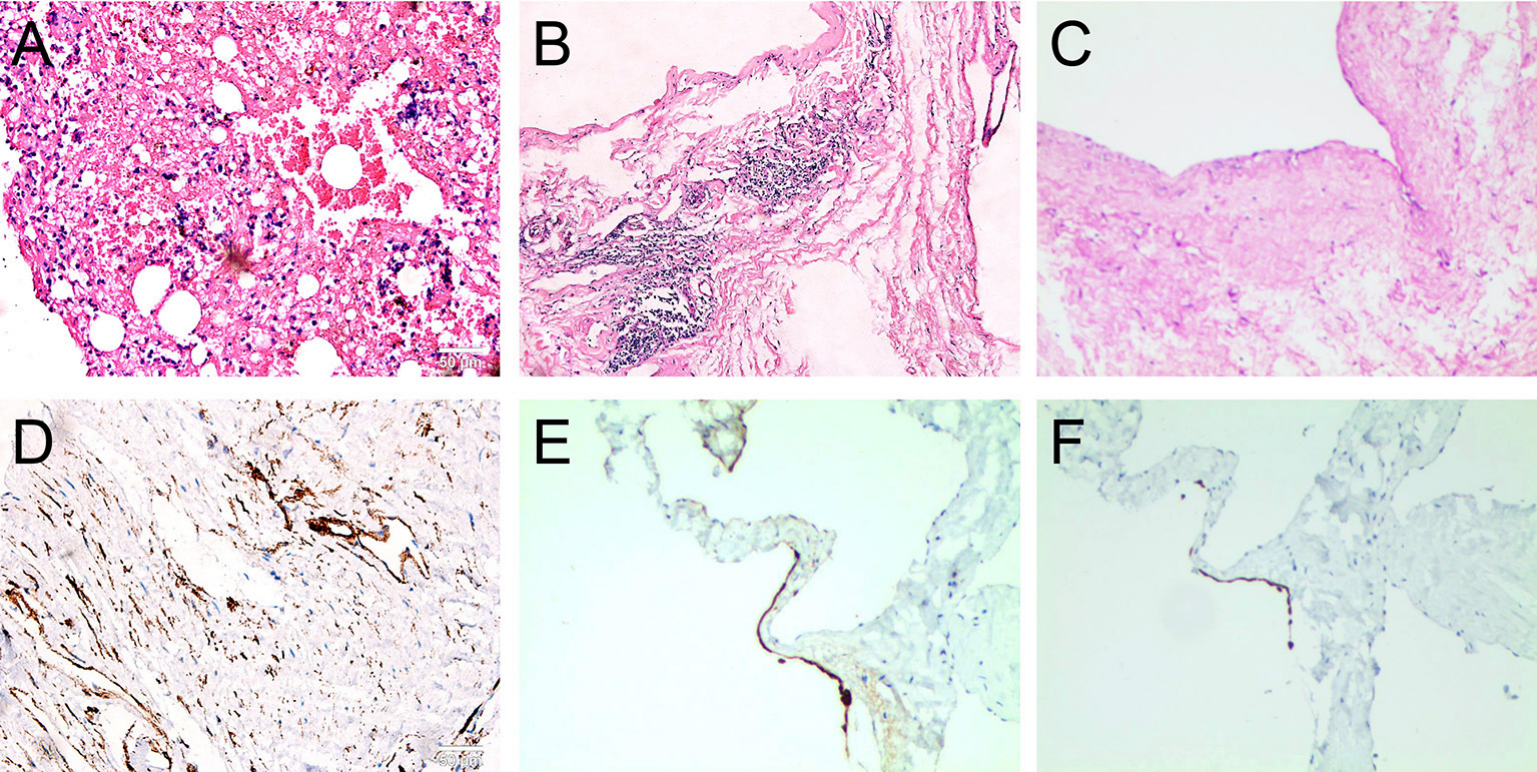
Post-operative histological examination with hematoxylin-eosin staining showing the proliferation of fibrous tissue in cyst wall **(A)**; hyalinosis **(B,C)**, lymphocyte infiltration **(B,C)**; and the immuohistochemical staining showing the positive expression of Ki-67, Vim, SMA, MC, and partial positive of D2-40 **(D)**, CK5/6 **(E)**, CR **(F)**, CD34, CD31, and WT-1, but negative of CK, EMA, MOC31, and Des (100X, scale bar = 50 μm).

**Figure 4:**
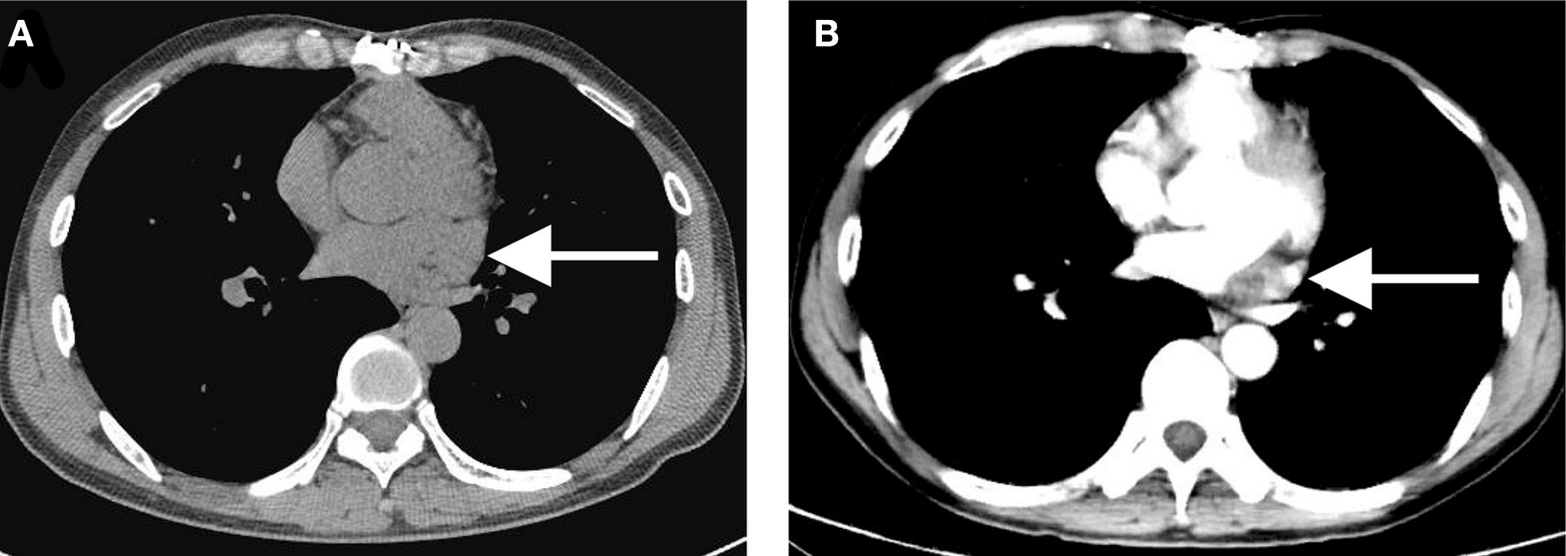
A 2- **(A)** to 4-year **(B)** post-operative computed tomography showing no evidence of remnant lymphangiohemangioma progression with the same size of 10 × 15 × 30 mm (arrow).

Notably, complete resection of the saccular structure could not be achieved following intraoperative findings; hence, partial resection of the mass was performed. The patient tolerated the procedure, and the heart was beating in sinus rhythm. After adequate hemostasis and closing the wound in layers, the patient was carefully transferred to the intensive care unit (ICU) in a stable condition. Pathological results of the surgically resected specimen confirmed the diagnosis of lymphangiohemangioma with the proliferation of fibrous tissue, hyalinosis, and lymphocyte infiltration. The results also indicated that the cells were positive for Ki-67, Vim, SMA, MC and partially positive for CR, CK5/6, CD34, CD31, D2-40, and WT-1, but negative for CK, EMA, MOC31, and Des (Figure 3). The patient recovered without complications and was discharged on the 7th post-operative day with a recommendation for follow-up. Cardiac CT 2- and 4-years post-surgery showed no evidence of remnant lymphangioma progression (Figure 4, arrow).

## Discussion

Primary cardiac tumors are sporadic, and they have often been diagnosed post-mortem (10). Pericardial lymphangiohemangiomas are sporadic benign tumors, with only one reported case in the literature (8). Cardiac lymphangioma is a benign cardiac tumor, and the symptoms appear only when the tumor size becomes large enough (11). Lymphangiohemangioma is a very rare slow-flow vascular malformation containing venous and lymphatic elements (7). Despite the advances in diagnostic techniques, including echocardiography, cardiac CT scans, FDG PET CT, and MRI, these lesions are most commonly diagnosed and treated surgically (8, 12). Given the increased risk of uncontrolled bleeding related to percutaneous biopsy, the definite diagnosis of pericardial lymphangiohemangioma is usually obtained with open surgical biopsy (1).

Nonetheless, the mass was generally unresectable in the case herein due to its significant invasion of surrounding tissues (1, 2, 8, 9). Thus, subtotal resection was the best surgical option given the clinical circumstances, which has been successfully adopted in the past. Previously, Robillard et al. reported a case of more than 8-years of follow-up study after a diagnostic surgical biopsy with a minimal increase in size. The patient reported occasional episodes of palpitation not consistent with arrhythmia (8).

Interestingly, Daubeney et al. report an intrapericardial lymphangioma with spontaneous regression 16-months after a pericardial window formation and biopsy of the mass (9). Given the structural type of the lymphangiohemangioma, we were unable to reach a definitive diagnosis. After a thorough discussion with the patient and his relatives, an exploratory median-sternotomy was performed. Operatively, the left atrium and ventricle's posterior wall were found to be infiltrated by the tumor. Due to the extreme rarity of the pericardial lymphangiohemangioma, no consistent guidelines for treatment and follow-up are presently available.

With the increased risk of bleeding and the lack of a pathologic diagnosis, we ruled it unsafe to attempt a total resection of the mass and instead carried out a partial resection. Notably, the patient's symptoms significantly improved after surgery. During a 4-years follow-up study of the patient, the tumor showed no evidence of progression nor regression, which was in contrast to the report by Robillard et al. (8) and Daubeney et al. (9). However, it is worth noting the short follow-up period of the study herein as there may be the possibility of relapse. Also, ample sample size and a sustained follow-up study are necessary to understand the long-term benefits and advantages of the procedure.

In conclusion, we report a rare case of primary pericardial lymphangiohemangioma located in the left atrioventricular groove, close to the left posterior atrial wall and the left ventricle. Subtotal resection of the tumor is a satisfactory method to relieve symptoms. To the best of our knowledge, the present case study is the first in the literature to report on the long-term post-operative outcome following subtotal resection of a pericardial lymphangiohemangioma.

## Data Availability Statement

The raw data supporting the conclusions of this article will be made available by the authors, without undue reservation.

## Ethics Statement

Written informed consent was obtained from the patient for publication of this case report and any accompanying images.

## Author Contributions

CF drafted the manuscript. CF and LS designed the study and responsible for the collection of data or analysis. LS and CD revised the manuscript. All authors read and approved the final manuscript.

## Conflict of Interest

The authors declare that the research was conducted in the absence of any commercial or financial relationships that could be construed as a potential conflict of interest.
